# Performance-improved thin-film a-Si:H/μc-Si:H tandem solar cells by two-dimensionally nanopatterning photoactive layer

**DOI:** 10.1186/1556-276X-9-73

**Published:** 2014-02-12

**Authors:** Cheng Zhang, Xiaofeng Li, Aixue Shang, Yaohui Zhan, Zhenhai Yang, Shaolong Wu

**Affiliations:** 1 Institute of Modern Optical Technologies & Collaborative Innovation Center of Suzhou Nano Science and Technology, Key Lab of Advanced Optical Manufacturing Technologies of Jiangsu Province & Key Lab of Modern Optical Technologies of Education Ministry of China, Soochow University, Suzhou 215006, China

**Keywords:** Tandem solar cells, Photonic crystal, Photocurrent matching

## Abstract

Tandem solar cells consisting of amorphous and microcrystalline silicon junctions with the top junction nanopatterned as a two-dimensional photonic crystal are studied. Broadband light trapping, detailed electron/hole transport, and photocurrent matching modulation are considered. It is found that the absorptances of both junctions can be significantly increased by properly engineering the duty cycles and pitches of the photonic crystal; however, the photocurrent enhancement is always unevenly distributed in the junctions, leading to a relatively high photocurrent mismatch. Further considering an optimized intermediate layer and device resistances, the optimally matched photocurrent approximately 12.74 mA/cm^2^ is achieved with a light-conversion efficiency predicted to be 12.67%, exhibiting an enhancement of over 27.72% compared to conventional planar configuration.

## Background

A common goal for photovoltaic (PV) design is to find effective ways to manage photons and excitons for high conversion efficiency by for example reducing cell reflection loss, improving light absorption of photoactive layers, and increasing charge collection [1]. The rapid progress of PV science has witnessed a lot of advanced light-trapping scenarios and technologies, such as impedance-matched coating [2], moth's eye structures [3], optical antennas [4], and photonic crystals [5]. Recent interests also focus on the applications of plasmonics in photovoltaics [6], e.g., by core-shell metallic nanowire design [7] or metallic gratings [8]. However, the strong parasitic absorption brings a big challenge to strictly balance the (negative) parasitic absorption loss and (positive) photocurrent gain of plasmonic solar cells (SCs) [9]. Therefore, conventional dielectric light-trapping structures are still attracting intensive research/application interests. Among these designs, photonic crystals are usually employed as an effective way to guide and confine the solar incidence, e.g., two-dimensional (2D) backside oxide grating [10] and low- or high-dimensional photonic structures [11,12].

The above designs are mainly dedicated to single-junction SCs. The strong demand for high photoconversion efficiency requires a more efficient use of the broadband solar incidence, leading to the generations of tandem and multi-junction cells. One important direction is the silicon-based tandem thin-film SCs (TFSCs), which are realized by introducing a layer of hydrogenated microcrystalline silicon (μc-Si:H) into conventional amorphous silicon (a-Si:H) SCs [13]. Compared to single-junction cells, a well-designed tandem solar cell has to be the combination of properly designed light trapping, efficient carrier transportation with low carrier loss, and perfectly matched photocurrent. Unlike the ordinary random texture or nanopattern in transparent conductive oxide (TCO), we recently proposed an a-Si:H/μc-Si:H tandem cell by nanopatterning the a-Si:H layer into one-dimensional (1D) grating. It is found that the realistic output photocurrent density (*J*_sc_) after current matching treatment can be greatly improved arising from a broadband absorption enhancement, which is stable against the changes of light polarization and injection direction [14].

Although under such a low-dimensional periodic design, a dramatic rise in photocurrent has been predicted in a purely optical means. It is thus reasonable to figure that further improvement could be possible by introducing a high-dimensional photonic crystal as it provides more controllable factors to optimize the PV behavior. Moreover, electrically evaluating the device response is necessary in order for a more accurate design on the tandem cells. In this paper, we first perform a thorough electromagnetic design based on rigorous coupled-wave analysis (RCWA) and finite-element method (FEM) for a-Si:H/μc-Si:H tandem TFSCs with a-Si:H layer nanopatterned as a 2D grating. Considering the dependence of the incident polarization and well engineering the key parameters of the 2D photonic crystal, we obtain the design with maximized absorption to the solar incidence. Our latest progress in simulating multi-junction SCs enables to look inside the microscopic charge behaviors of the a-Si:H/μc-Si:H tandem cells so that the electrical response as well as the photocurrent matching degree of the SCs from optical design can then be evaluated in a precisely electrical way. To match the photocurrents between the junctions, a modified design with an intermediate layer is proposed. The optimized cell exhibits light-conversion efficiency up to 12.67%, which is enhanced by 27.72% over its planar counterpart.

## Methods

**Figure 1:**
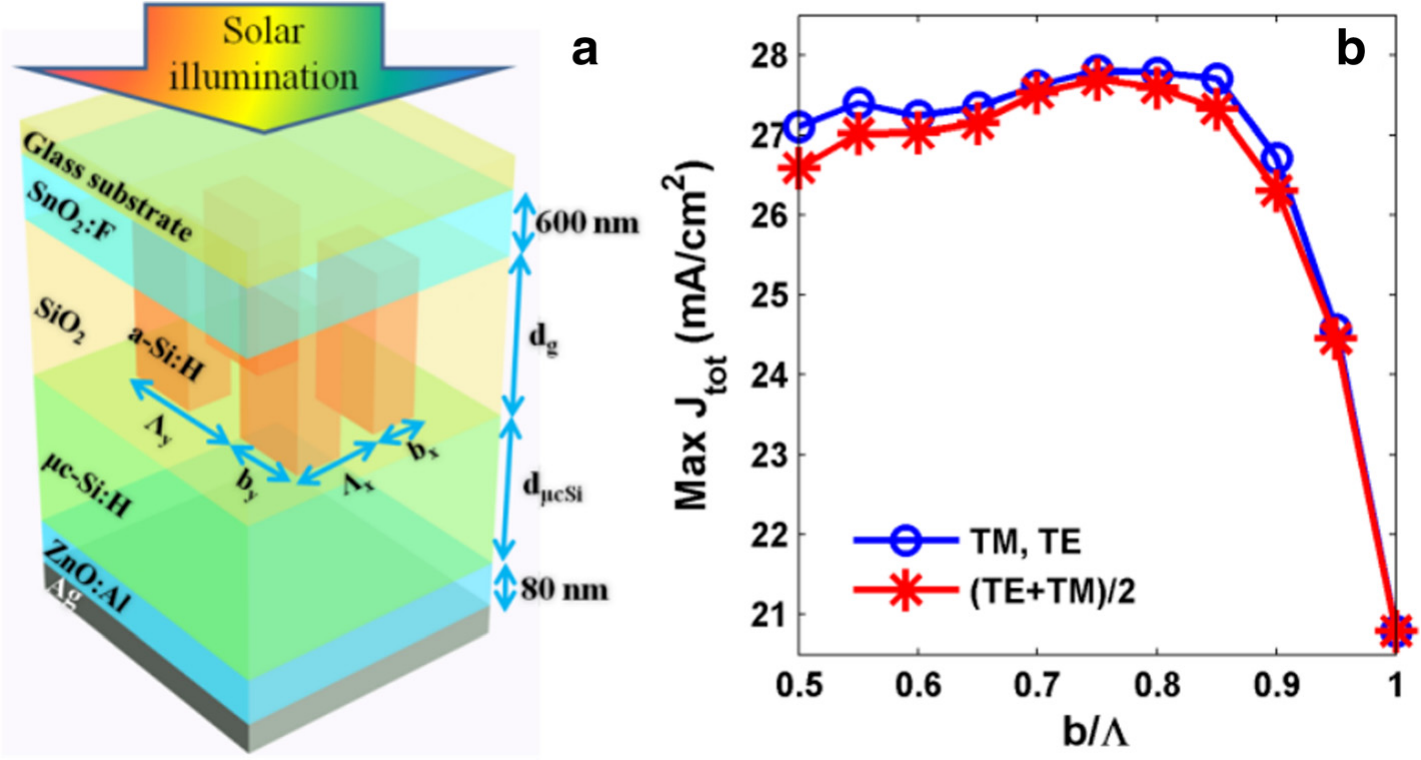
**Device and duty cycle optimization. (a)** Schematic diagram of a-Si:H/μc-Si tandem TFSCs with a-Si:H layer nanopatterned into 2D grating; **(b)** maximal total current, max(*J*_tot_), as a function of duty cycle (*b*/*Λ*).

Figure 1a shows the diagram of the considered tandem TFSC under a superstrate configuration, which is composed of the glass substrate, SnO_2_:F top TCO, a-Si:H top junction grated by SiO_2_, μc-Si:H bottom junction, ZnO:Al bottom TCO, and rear silver (Ag) reflector. *Λ*_*x*_ (*Λ*_*y*_) and *b*_*x*_ (*b*_*y*_) are the pitch and grating width along *x* (*y*) direction, respectively, and *d*_g_ is the grating depth. The thicknesses of top and bottom TCOs are 600 and 80 nm, respectively, in order to ensure a satisfactory device conductivity. For the convenience of photocurrent match, we assume a planar system with the thickness of a-Si:H (*d*_aSi_) [μc-Si:H layers (*d*_ucSi_)] to be 220 nm (1,700 nm). The PV materials are with fixed volumes under various nanodesigns, i.e., for a-Si:H layer *d*_aSi_*Λ*_*x*_*Λ*_*y*_ = *b*_*x*_*b*_*y*_*d*_*g*_, ensuring a fair evaluation of the device performance.

Most optical simulations in this study are based on 2D RCWA, which considers the periodicities along both *x* and *y* directions and thus is very applicable for analyzing high-dimensionally periodic structures. To make sure the accuracy and reduce the time of computation, the first 11 diffraction modes are taken into account. It is especially useful for performing optimization task for periodic three-dimensional (3D) nanosystems through wide-range parametric sweep. However, RCWA does not give the full information for SCs, especially for those composed by multiple PV layers. Nevertheless, distinguishing the contribution from each PV layer is crucial for tandem SCs in order to score the photocurrent matching degree. Therefore, a complementing full-wave FEM method is used to obtain the detailed absorption information for the selected systems after initial RCWA designs. The meshes are chosen carefully according to the routine that the maximum element size being no greater than min(*λ*)/10/max[*n*(*λ*)], where *λ* is the concerned wavelength and *n*(*λ*) is the wavelength-dependent refractive index. For a-Si and μc-Si, we adopt the optical database from [15]; while for Ag and ZnO, the optical constants are from Palik [16]. Since *p* and *n* regions considered are lightly doped, along with their thin thicknesses (tens of nanometers), the semiconductor doping can be deemed to bring neglectable effect on the optical absorption. FEM calculation also demonstrates that (1) the absorption of top TCO is stable under various configurations and (2) the bottom TCO absorption is very weak because the short-wavelength light has almost been depleted completely before reaching the bottom. For these reasons, the photoactive absorption (*P*_abs_) can be obtained by eliminating the top TCO absorption from the total absorption calculated from RCWA, and the total photocurrent *J*_tot_ is then predicted roughly from *P*_abs_ under the assumption of perfect internal quantum process.

The above optical treatment can reflect the total absorption and overall photocurrent characteristics of the tandem SCs to some extent. However, perfect carrier transportation is generally not possible. A realistic device-oriented simulation for SCs requires performing an optical-electrical simulation by connecting the electromagnetic and carrier transport calculations simultaneously (see [9,17,18] for details). For the tandem cells, we need the optical-electrical simulations for both top and bottom junctions with carrier generation, recombination, transport, and collection mechanisms totally included. The carrier generation profile in each junction is from the electromagnetic calculation. This way, the actual external quantum efficiencies (EQEs) and short-circuit photocurrent densities (*J*_aSi_ and *J*_μcSi_) of the two junctions can be achieved, yielding the *J*_sc_ = min(*J*_aSi_, *J*_μcSi_). With the dark current response calculated [18], we can construct the current–voltage (*J*-*V*) curve for the tandem TFSCs and carefully evaluate the cell performance, such as open-circuit voltage (*V*_oc_) and light-conversion efficiency (*η*) under various nanophotonic designs.

## Results and discussion

As the featured size of the nanopattern is comparable to the wavelength, the strong light-matter interaction is extremely sensitive to the geometric configurations, providing an efficient way of controlling sub-wavelength light-trapping behaviors. In this study, the integrated absorption is determined by the key parameters of the 2D grating, i.e., the height (*d*_*g*_), pitches (*Λ*_*x*_, *Λ*_*y*_), and widths (*b*_*x*,_*b*_*y*_). Two-dimensional RCWA facilitates to find the optimized total photocurrent *J*_tot_ (= *J*_aSi_ + *J*_μcSi_) by properly designing *Λ* and duty cycle *b*/*Λ* in both directions. Under a perfect internal quantum process, the upper limit of total photocurrent (*J*_tot_) is obtained by integrating spectrally the absorption *P*_abs_ (which has excluded the absorptions from non-photoactive layers revised by FEM [14]) over the band of 300 ≤ *λ* ≤ 1,100 nm weighted by the standard AM 1.5 spectra [19].

The plot in Figure 1b illustrates the max(*J*_tot_) versus *b*/*Λ* (*b*_*x*_*/Λ*_*x*_ = *b*_*y*_*/*Λ_y_). It should be noted that although only *b*/*Λ* is given in the figure, the results are actually from a number of 2D parametrical sweep for both *Λ* (from 300 to 1,100 nm with step 50 nm) and *b*/*Λ* (from 0.5 to 1 with step 0.05), i.e., the 3D PV system has been simulated for hundreds of times in order to find the designs with the highest *J*_tot_. For each *b*/*Λ*, only the maximized *J*_tot_ under an optimized *Λ*, which generally varies under different *b*/*Λ*, is recorded. Compared to the planar cell (i.e., *b*/*Λ* = 1) with *J*_tot_ approximately 20.79 mA/cm^2^, two-dimensionally nanopatterning top junction always leads to a much higher *J*_tot_ with a peak of 27.69 mA/cm^2^ (see red curve for unpolarized case) at *b*/*Λ* = 0.75, *Λ*_*x*_ = 450 nm, and *Λ*_*y*_ = 850 nm. In addition, transverse electric (TE, i.e., electrical field *E* along *y*) and transverse magnetic (TM, i.e., *E* along *x*) incidences show identical max(*J*_tot_) due to the geometrical symmetry, while the value for unpolarized, i.e., (TE + TM)/2, is generally lower.

**Figure 2:**
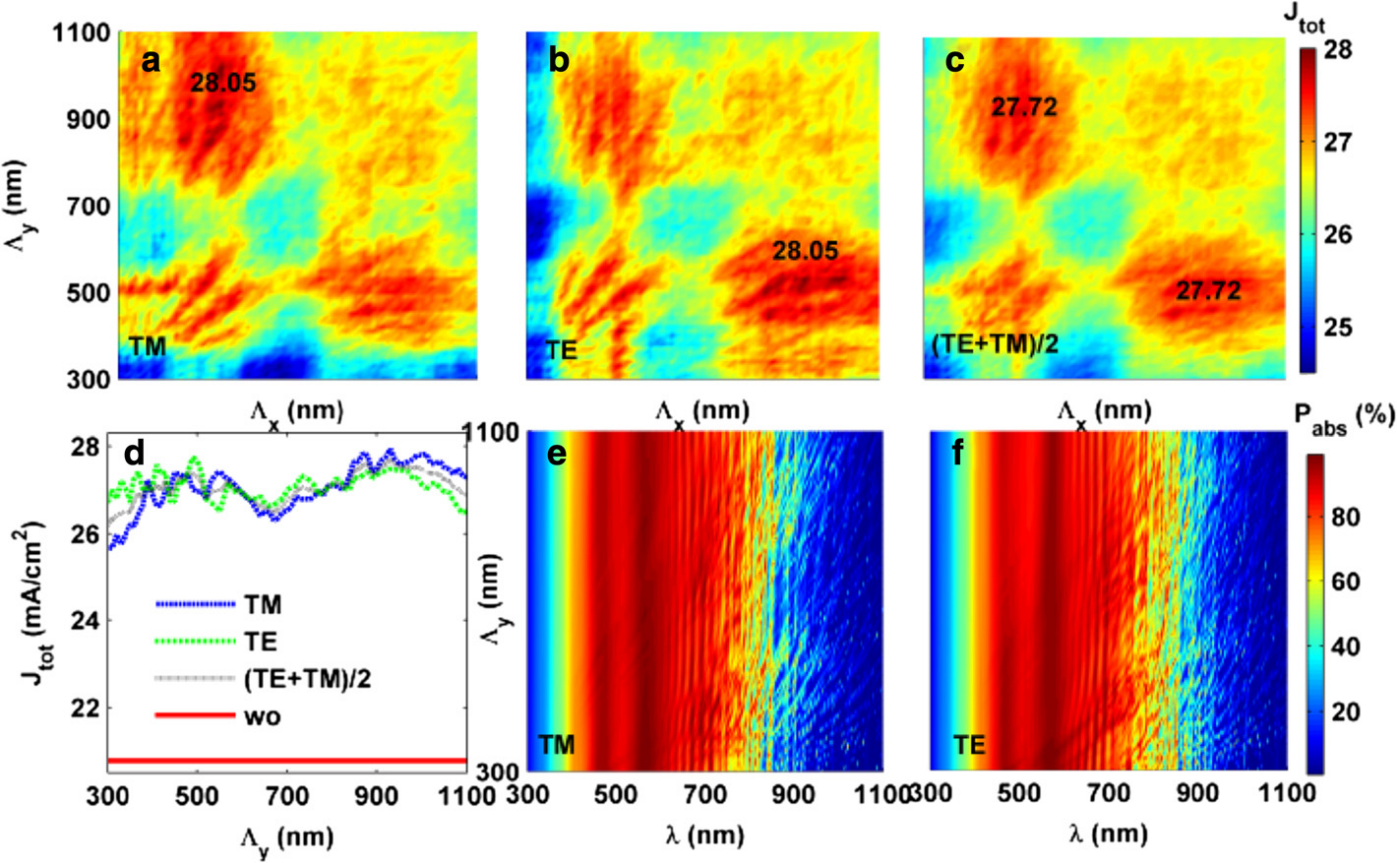
**Grating pitch optimization and absorption spectra. ***J*_tot_ versus *Λ*_*x*_ and *Λ*_*y*_ for **(a)** TM, **(b)** TE, and **(c)** (TE + TM)/2; **(d)***J*_tot_ versus *Λ*_*y*_ at *Λ*_*x*_ = 520 nm with planar case (wo, i.e., without nanopattern design) for reference; *P*_abs_ versus *Λ*_*y*_ and *λ* under **(e)** TM and **(f)** TE incidences, where *Λ*_*x*_ = 520 nm. *b*/*Λ* = 0.75 (according to Figure 1) is used in all figures.

To explore the physics behind the above observation, contour maps of max(*J*_tot_) versus *Λ*_*x*_ and *Λ*_*y*_ are given in Figure 2a,c for TM, TE, and unpolarized cases, respectively. In these figures, *b*/*Λ* = 0.75 is used according to the design of Figure 1 and the peaked *J*_tot_ values in mA/cm^2^ have been marked directly. Comparing Figure 2 panels a and b, the photocurrent maps for TE and TM cases are mutually symmetrical with respect to the line of *Λ*_*y*_ = *Λ*_*x*_. This is rational since it is completely equivalent to rotate either the electric polarization or the device by 90° in the *x*-*y* plane. This answers the question that why the curves (in blue) for TE and TM are undistinguishable in Figure 1b. However, *J*_tot_ is not peaked under the same grating pitches for TE or TM (see Figure 2a,b). A direct consequence is that the maximal *J*_tot_ for unpolarized illumination cannot reach the value under linear polarization. This can be seen from Figure 2c, where max(*J*_tot_) = 27.72 mA/cm^2^ (<28.05 mA/cm^2^ from linear case) is found at *Λ*_*x*_ = 520 nm and *Λ*_*y*_ = 930 nm. It should be noted that the peaked value and optimal pitches are slightly changed from Figure 1b since a finer sweep with *Λ* step of 10 nm is employed.

Figure 2d plots *J*_tot_ as a function of *Λ*_*y*_ with *b*/*Λ* = 0.75 and *Λ*_*x*_ = 520 nm for all interested polarizations conditions. Also inserted is the *J*_tot_ of the planar system mentioned previously. A photocurrent enhancement approximately 6 mA/cm^2^ is achieved under most of grating designs with both linearly polarized and unpolarized incidences, showing the attractiveness of the new tandem TFSCs with a 2D nanopatterned photoactive layer. Since top TCO is considered in this paper to be with 600 nm for electrical consideration unlike what we used in [14], a complete 1D nanopattern design similar to [14] is also performed. Optimized 1D design yields *J*_tot_ = 24.49 mA/cm^2^, which is apparently lower than that under 2D nanophotonic configuration (i.e., *J*_tot_ approximately 27.72 mA/cm^2^ with an increment of 3.23 mA/cm^2^). This arises from the fact that more solar energy is coupled two-dimensionally into the resonant modes in the a-Si:H/μc-Si active layers under a light-trapping mechanism with 2D photonic crystal [6]. Figure 2e,f is the (overall) absorption spectra (*P*_abs_) of the tandem TFSCs under various *Λ*_*y*_. It is obvious that the tandem cell has very good light absorption performance (except that absorbed by top TCO when *λ* < 400 nm) in the active band, especially within the band of 400 < *λ* < 700 nm.

**Figure 3:**
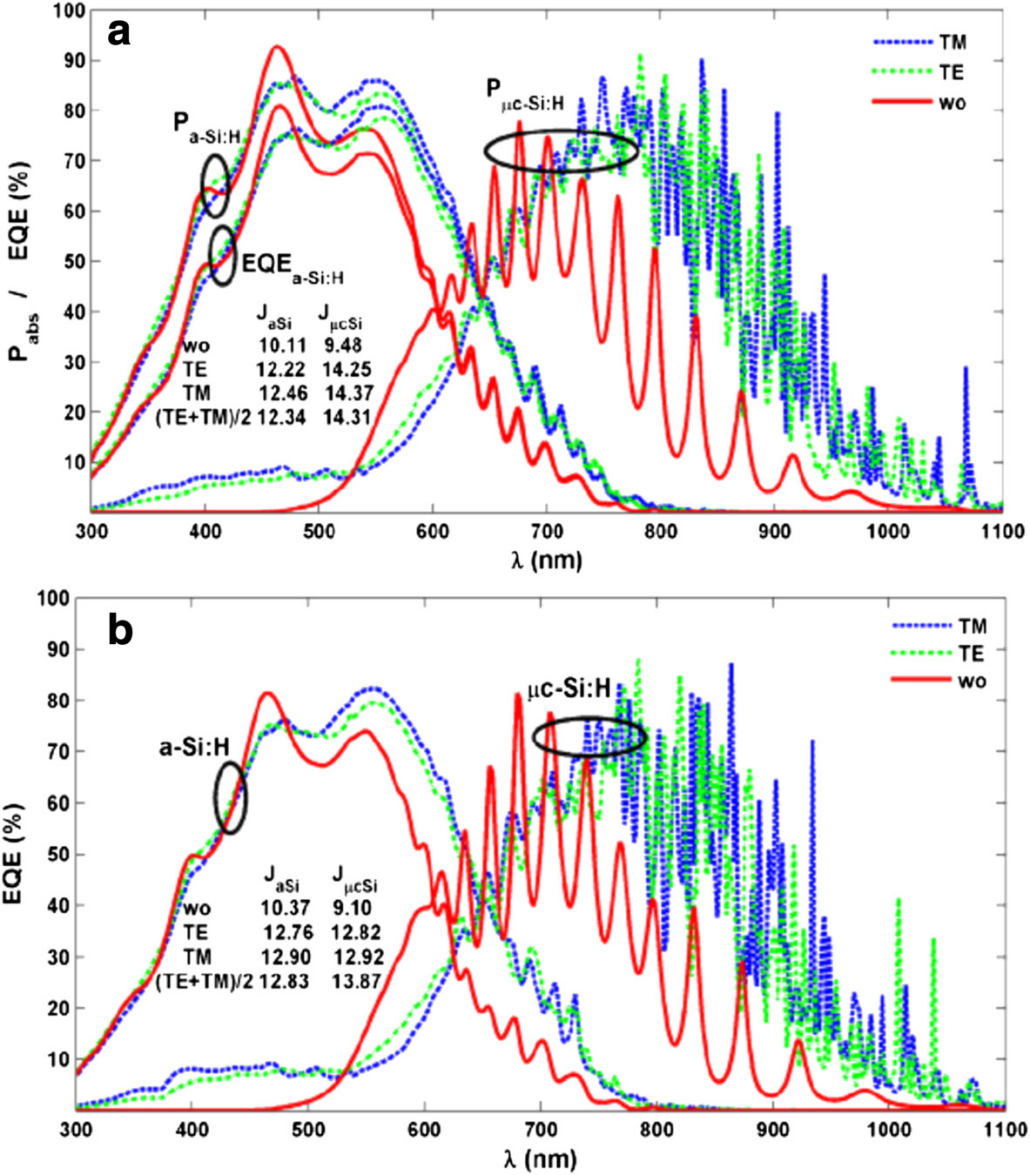
**EQE spectra. ***P*_abs_ and EQE spectra of a-Si:H/μc-Si tandem TFSCs with *b*/*Λ* = 0.75, *Λ*_*x*_ = 520 nm, and *Λ*_*y*_ = 930 nm, where a 18-nm ZnO layer is sandwiched by two junctions in **(b)** (noted: no ZnO layer in **(a)**). In Figures 3 and 4, ellipses are used to categorize the simulation results.

For the optimized design (*b*/*Λ* = 0.75, *Λ*_*x*_ = 520 nm, and *Λ*_*y*_ = 930 nm) from 2D RCWA, we turn to FEM calculation in order to get the detailed absorption distributions in the tandem junctions. Absorption spectra for a-Si:H and μc-Si:H layers (i.e., *P*_a-Si:H_ and *P*_μc-Si:H_) are plotted in Figure 3a, where TE, TM, unpolarized, and planar (wo) cases are considered. Compared to the 1D grating design [14], nanopatterning a-Si:H layer into 2D grating further improves the junction capability of harvesting the solar energy. Especially, *P*_μc-Si:H_ under either TE or TM incidence is dramatically strengthened, e.g., *P*_abs_ = 71.61% for TE (5.402% for wo) at *λ* = 886 nm and 79.85% for TM (5.121% for wo) at 902 nm. In addition, there are much more resonant peaks in the spectrum due to the strong cavity effects and the presence of a great deal of diffraction modes excited from the 2D grating. This can be very beneficial to realize a broadband absorption enhancement. For the top junction, 2D grating also improves the light absorption than 1D case, resulting in a maximized *J*_tot_ as discussed previously.

To evaluate the electrical response of each junction, a device simulation which couples both optical absorption and carrier transport are performed [17,18]. P/i/n setup is assumed for both junctions with p/n doping concentration of 1.3 × 10^17^/4.3 × 10^16^ cm^−3^ and thickness of 10/30 nm (the rest is intrinsic region). Electron (hole) mobility in p/i/n region for top junction is 4.6/4.6/100 (50/0.92/0.92) × 10^−6^ m^2^/V/s [17] and carrier mobility 100 times over those in top junction are used for the μc-Si:H junction. Carrier lifetime is from [17], and the surface recombination coefficient is 1 × 10^2^ (1 × 10^4^) cm/s for interior (external) interfaces.

Figure 3a reveals that the imperfect internal quantum process caused by the surface recombination and other carrier loss mechanisms results in a great degradation on the electrical properties of the top (a-Si:H) cell, which is reflected as a much discrepancy between *P*_a-Si:H_ and EQE_a-Si:H_ especially at short-wavelength region. However, for the bottom junction, *P*_μc-Si:H_ ~ EQE_μc-Si:H_ is always observed since the material defects are much less and the bottom junction is far from the top surface where the surface recombination is strong. Spectral integrations to the EQE spectra indicate that under TE (TM) illumination, *J*_aSi_ can be risen by 2.11 (2.35) mA/cm^2^, resulting in the rise of 2.23 mA/cm^2^ in the top junction under an unpolarized injection. However, the raise of photocurrent in bottom junction is especially dramatic (4.63 mA/cm^2^), which has been actually expected from the multi-peaked absorption spectra. Therefore, although significant improvement on the absorption and light-conversion capability has been realized by two-dimensionally nanopatterning a-Si:H. The performance gain has not been evenly distributed to the top and bottom junctions, leading to a photocurrent mismatch high up to 2 mA/cm^2^.

It is found that the incorporation of a ZnO intermediate layer between the junctions can increase the absorption and photocurrent of the top junction through light reflection from the a-Si:H/ZnO/μc-Si:H interfaces [13]. However, a too thick ZnO layer leads to rapidly degraded total photocurrent; therefore, its thickness has to be designed carefully. According to our calculation, a ZnO layer with thickness of 18 nm is an optimal design for realizing the best photocurrent match without degrading *J*_tot_ noticeably. EQE spectra of a-Si:H and μc-Si:H junctions incorporating the intermediate ZnO layer are given in Figure 3b. Comparing to Figure 3a, it can be seen that for wavelength between 500 and 700 nm, the EQE_a-Si:H_ has been increased for a higher *J*_aSi_. Since less light is coupled into μc-Si:H layer, *J*_μcSi_ is slightly lowered for better current match. By integrating 2D nanopattern and ZnO intermediate designs into the a-Si:H/μc-Si:H tandem TFSCs, *J*_sc_ can be up to 12.83 mA/cm^2^ under an unpolarized solar illumination, which has been enhanced by 35.34% compared to the planar system (i.e., increases by 3.35 mA/cm^2^ from 9.48 mA/cm^2^).

**Figure 4:**
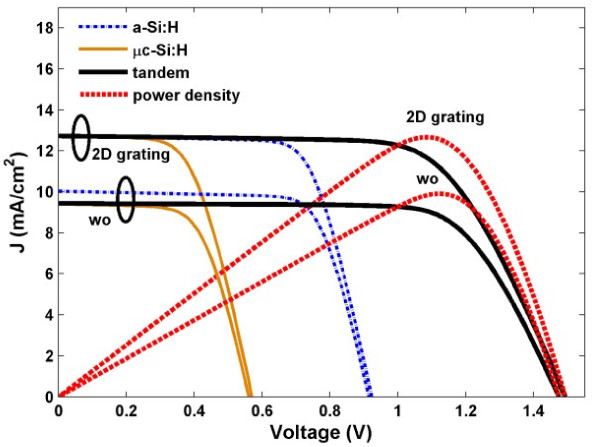
***J*-*V *characteristic of the a-Si:H top cell, μc-Si:H bottom cell, and a-Si:H/μc-Si:H tandem cell.** Power densities versus *V* are also inserted for the designed tandem cell and reference cell.

Finally, based on the previously calculated *J*_sc_ and the dark current densities in top and bottom junctions under continuously increasing forward electric biases (V), the current–voltage characteristics of the proposed a-Si:H/μc-Si tandem TFSCs obtained are explored and illustrated in Figure 4. For an accurate prediction of the electrical performance, series and shunt resistances (*R*_s_ and *R*_sh_) of the solar devices have been taken into account. The values of *R*_s_ and *R*_sh_ can be obtained by extracting the slope information from the *J*-*V* curve at the points of *V* = 0 and *V*_oc_, respectively [20]. In this study, the experimentally measured *J*-*V* curve from [21] is used due to the similar device configuration. The calculated *R*_s_ and *R*_sh_ are 10 and 2,800 Ω · cm^2^, respectively. From the illustration, performance parameters like maximum output power density (*P*_max_), *V*_oc_, fill factor [*FF* = *P*_max_/(*J*_sc_V_oc_)], and *η* can be obtained. It is found that the tandem configuration can achieve a much higher *V*_oc_ approximately 1.5 V, which does not change much under various light-trapping designs. However, *J*_sc_ shows great increase under the optimal 2D photonic crystal design, leading to a much higher *P*_max_. Under a FF approximately 66.75%, *η* = 12.67% is predicated with an enhancement ratio of 27.72% compared to the reference.

## Conclusions

a-Si:H/μc-Si:H tandem TFSCs with improved absorption and light-conversion efficiency are presented in this paper. Full-wave electromagnetic and detailed carrier transport calculations are used for a thorough design on the optical and electrical performance of the nanostructured tandem SCs. The maximized photocurrent matched between two junctions is realized by two-dimensionally nanopatterning a-Si:H top junction into 2D photonic crystal and introducing an optimized intermediate layer between the junctions. Considering both optical and electrical perspectives, a tandem cell with a relative increase of 35% (27.72%) in *J*_sc_ (*η*) can be achieved under the optimized photonic design. Compared to conventional tandem cell in 1D nanopattern, the proposed system exhibits an improved light absorbing and conversion capability due to the better confinement to the solar incidence under strong diffraction and waveguiding effects, and therefore it is believed to be a promising way of realizing high-efficiency tandem TFSCs. Finally, we would like to indicate that the designed system is with typical 2D grating structure, which has been extensively used in various optoelectronic fields and can therefore be fabricated by standard nanofabrication methods, including optical (sometimes electrical) lithography, nanoimprinting, or laser holographic lithography [22,23]. The fabrication of a-Si:H/μc-Si:H tandem TFSC can be found from literatures (e.g., [24]).

## Abbreviations

1D: one-dimensional; 2D: two-dimensional; 3D: three-dimensional; EQEs: external quantum efficiencies; FEM: finite-element method; FF: fill factor; J-V: current–voltage; PV: photovoltaic; RCWA: rigorous coupled-wave analysis; SCs: solar cells; TCO: transparent conductive oxide; TE: transverse electric; TFSCs: thin-film solar cells; TM: transverse magnetic.

## Competing interests

The authors declare that they have no competing interests.

## Authors’ contributions

CZ carried out the design and drafted the manuscript. XL conceived the design and supervised the research. AS and ZY participated in the *J*-*V* simulation. YZ and SW commented on the results and revised the manuscript. All authors read and approved the final manuscript.
